# Sox2 suppresses the invasiveness of breast cancer cells via a mechanism that is dependent on Twist1 and the status of Sox2 transcription activity

**DOI:** 10.1186/1471-2407-13-317

**Published:** 2013-07-01

**Authors:** Fang Wu, Xiaoxia Ye, Peng Wang, Karen Jung, Chengsheng Wu, Donna Douglas, Norman Kneteman, Gilbert Bigras, Yupo Ma, Raymond Lai

**Affiliations:** 1 Department of Laboratory Medicine and Pathology, University of Alberta, Edmonton, Alberta, Canada; 2 Department of Oncology, University of Alberta, Edmonton, Alberta, Canada; 3 Department of Surgery, University of Alberta, Edmonton, Alberta, Canada; 4 Department of Pathology, Stonybrook University, Stonybrook, NY, USA; 5 DynaLIFEDX Medical Laboratories, Edmonton, Alberta, Canada

**Keywords:** Sox2, Transcription activity, Invasiveness, Twist1, Breast cancer

## Abstract

**Background:**

Sox2, an embryonic stem cell marker, is aberrantly expressed in a subset of breast cancer (BC). While the aberrant expression of Sox2 has been shown to significantly correlate with a number of clinicopathologic parameters in BC, its biological significance in BC is incompletely understood.

**Methods:**

*In-vitro* invasion assay was used to evaluate whether the expression of Sox2 is linked to the invasiveness of MCF7 and ZR751 cells. Quantitative reverse transcriptase-polymerase chain reaction (qRT-PCR) and/or Western blots were used to assess if Sox2 modulates the expression of factors known to regulate epithelial mesenchymal transition (EMT), such as Twist1. Chromatin immunoprecipitation (ChIP) was used to assess the binding of Sox2 to the promoter region of *Twist1*.

**Results:**

We found that siRNA knockdown of Sox2 expression significantly increased the invasiveness of MCF7 and ZR751 cells. However, when MCF7 cells were separated into two distinct subsets based on their differential responsiveness to the *Sox2* reporter, the Sox2-mediated effects on invasiveness was observed only in ‘reporter un-responsive’ cells (RU cells) but not ‘reporter responsive’ cells (RR cells). Correlating with these findings, siRNA knockdown of Sox2 in RU cells, but not RR cells, dramatically increased the expression of Twist1. Accordingly, using ChIP, we found evidence that Sox2 binds to the promoter region of *Twist1* in RU cells only. Lastly, siRNA knockdown of Twist1 largely abrogated the regulatory effect of Sox2 on the invasiveness in RU cells, suggesting that the observed Sox2-mediated effects are Twist1-dependent.

**Conclusion:**

Sox2 regulates the invasiveness of BC cells via a mechanism that is dependent on Twist1 and the transcriptional status of Sox2. Our results have further highlighted a new level of biological complexity and heterogeneity of BC cells that may carry significant clinical implications.

## Background

Tumor invasiveness is a complex process in which malignant cells dissociate and migrate from the primary site of growth, which may eventually lead to the formation of distant metastases [1]. In many types of solid tumor, it has been shown that epithelial-mesenchymal transition (EMT) is a crucial step for tumor invasiveness [2,3]. During EMT, malignant epithelial cells shed their differentiated characteristics (e.g. cell-cell adhesion, apical-basal polarity and immobility) and acquire mesenchymal features (e.g. increased motility and invasiveness) [4]. The induction of EMT can be triggered by cytokines, such as TGF-β and interleukin (IL)-8, as well as several transcriptional factors including Twist1, Snail, and ZEB [5-9]. Twist1 has been described to be one of the key promoters of EMT and invasiveness in a number of cancer types [10-12]. In several studies, Twist1 was found to be up-regulated by a number of proteins including STAT3 [13], BMP2 [14], SRC-1 [15], MSX2 [16], NF-κB [17], and ILK [18] and down-regulated by miR-580 and CPEB1/2 [19]. In breast cancer (BC), Twist1 has been found to promote EMT and invasiveness [5]. A number of immunohistochemical studies have described a significant positive correlation between Twist1 and the metastatic/invasive property of BC [5-8]. In an animal model, siRNA knockdown of Twist1 was found to inhibit BC cells to metastasize to the lungs [5]. Furthermore, the mechanisms by which Twist1 promotes tumor invasiveness in BC have been extensively examined; down-regulation of E-cadherin [9] and up-regulation of SET8 [20], AKT2 [8], miRNA-10b [21], IL8 [22] and PDGFα [23] have been implicated.

Sox2 (sex determining region Y-box protein 2) is a transcription factor that plays a key role in maintaining the pluripotency of embryonic stem cells [24-26]. The importance of Sox2 in stem cell biology is highlighted by the fact that Sox2 represents one of the 4 genes implicated in the conversion of fibroblasts into inducible pluripotent stem cells [27,28]. Recent studies have shown that Sox2 is aberrantly expressed in several types of solid tumors, including BC, lung cancer, prostate cancer, glioblastomas and melanomas [29-33]. The expression of Sox2 detectable by immunohistochemistry has been found to positively correlate with the invasiveness and metastatic potential of several types of solid tumors [34-37]. Nevertheless, *in-vitro* studies that directly assess the role of Sox2 in regulating tumor invasiveness are relatively scarce [35-38]. In several types of cancer cells (e.g., gliomas, melanomas and colorectal cancer), knockdown of Sox2 using siRNA was found to decrease invasiveness [35-37]. In one study, enforced expression of Sox2 in MCF7, an estrogen receptor-positive (ER+) BC cell line, was found to increase invasiveness in an *in-vitro* assay by approximately 60% [38]. The mechanisms by which Sox2 regulates the invasiveness of BC cells are largely unknown. For instance, whether the regulatory effects of Sox2 on the invasiveness of BC are linked to regulators of EMT (such as Twist1) has not been examined previously.

In this study, we aimed to further define the roles of Sox2 in regulating the invasiveness of BC cells. In contradiction with the conclusion of a recently published paper [38], we found that Sox2 suppresses, rather than increases, the invasiveness of MCF7 cells. Furthermore, this biological effect is dependent on the regulation of Twist1 expression by Sox2. When we assessed the roles of Sox2 in the two distinct cell subsets of MCF7 separated based on their differential responsiveness to the *Sox2* reporter, as shown previously [39], we found that the Sox2-mediated effects on invasiveness in BC is restricted to ‘reporter un-responsive’ (RU) cells. We believe that our results have shed important insights into the biological significance of Sox2 in BC, the invasiveness property of BC, as well as a new level of biological complexity of this type of cancer.

## Methods

### Cell culture

MCF7 and ZR751 were purchased from American Type Culture Collection (ATCC, Rockville, MD). Both ZR751 and MCF7 cells were maintained in high glucose Dulbecco's Modified Eagle Medium (DMEM) (Life Technologies, Grand Island, NY) supplemented with 10% fetal bovine serum (FBS) (Sigma, Oakville, ON, Canada) and were cultured under an atmosphere of 5% CO_2_ at 37°C.

### Generation of stable cell lines

Stable cells expressing the *Sox2 GFP* reporter were generated as previously described [39]. Cells stably expressing the *Sox2 GFP* reporter were cultured in DMEM, supplemented with 10% FBS, 100 U/ml penicillin, 100 ng/ml streptomycin. 1 μg/ml of puromycin was added to the culture medium at all times. The generated stable cell clones were analyzed for GFP expression by flow cytometry every two weeks over a 4-month period. RR and RU cells were sorted out based on GFP expression and cultured separately. The two populations remained 98% pure over 4 months.

### Gene silencing

MCF7 and ZR751 cells were transfected with 1 nmol of SMARTpool siRNA designed against Sox2 (Thermo Scientific). Scramble non-targeting siRNA (Thermo Scientific) was used as the negative control. For all siRNA transfection, a BTX 830 electroporation instrument (Harvard Apparatus, Holliston, MA) was used. For double knockdown experiments, SMARTpool siRNA designed against Twist1 from Thermo Scientific was used.

Enforced expression of Sox2 in MCF7 cells was performed as previously described [39]. Briefly, pheonix packaging cells were transfected with either pMXs *Sox2* retroviral vector (Addgene, MA, USA) or empty vector according to the manufacturer's suggestion. MCF7 cells were infected with retroviral particles three times in 24 hour intervals. 48 hours after the final infection, cells were overnight starved and were then used to perform invasion assay.

### Western blotting

Western blot analyses were performed as previously described [40,41]. The following antibodies were used: Sox2 (Cell Signaling Technologies), Twist1 (Santa Cruz), γ-Tubulin (Sigma).

### Cell viability

Cell viability was determined using the 3-(4,5-dimethylthiazol-2-yl)-5-(3-carboxymethoxyphenyl)-2-(4-sulfophenyl)-2H-tetrazolium, inner salt (MTS) assay (Promega, Madison, WI) according to the manufacturer's protocol.

### Cell invasion assay

As previously described, we assessed cell invasiveness using the Cytoselect™ 24-well cell invasion assay kit (Cell Biolabs, San Diego, CA, USA) according to the manufacture’ s protocol [42]. Briefly, cells were overnight starved prior to invasion assay. Approximately 1 × 10^5^ cells in serum free medium were plated in the top chamber and medium supplemented with 10% FBS was used as a chemo-attractant in the lower chamber. The cells were then allowed to invade the reconstituted basement membrane matrix for 24 hours. The invasive cells passed the membrane were then dissociated from membrane, lysed and quantified using CyQuant GR fluorescent Dye.

### Quantitative RT-PCR

Total RNA was extracted using TRIzol according to the manufacturer’s protocol. Quantitative RT-PCR was performed using Applied Biosystem Prism 7900HT instruments. The TaqMan gene expression assay (Applied Biosystems) used were: Hs01548727_m1 (*MMP2),* Hs00234579_m1 *(MMP9),* Hs01675818_s1 *(Twist1),* Hs01023894_m1 *(E-cadherin), Hs00362037_m1 (N-cadherin, Hs00232783_m1(ZEB1) and* Hs00998133_m1 *(TGF-β).* Primer sequences for *Snail* are: Forward 5'-acaaaggctgacagactcactg-3′, Reward 5′-tgacagccattactcacagtcc-3′. Primer sequences for *Slug* are: Forward 5′-gtctctcctgcacaaacatgag-3′, Reverse 5′-atgctcttgcagctctctctct-3′. Primer sequences for *MMP3*: Forward 5′-cactcacagacctgactcggtt-3′, Reverse 5′- aagcaggatcacagttggctgg-3′. Primer sequence for *FAK* are Forward 5′-gccttatgacgaaatgctgggc-3′, Reverse 5′- cctgtcttctggactccatcct -3′. Human *GAPDH* was used as control. Expression of each gene was measured in triplicate.

### Chromatin immunoprecipitation (ChIP) assay

ChIP assay was performed as our previously described [39]. The chromatin was extracted from MCF7-RR and -RU cells. A normal rabbit IgG antibody and anti-Sox2 antibody (Santa Cruz) was then incubated with the chromatin. Isolated DNA was then amplified with *Twist1* primers (−1478 to −1322 of transcriptional start site, 156 bp amplicons): Forward 5′-ggcgagtccgtactgagaag-3′ Reverse 5′- cgtttcaggtccatccctta-3′.

### Statistical analysis

All the statistical analyses were performed using the GraphPad Prism 5.1 program. Student T-test and One-way ANOVA were used to calculate p value. Results are presented as mean ± standard deviation.

## Results

### Sox2 suppresses the invasiveness of breast cancer cells

**Figure 1:**
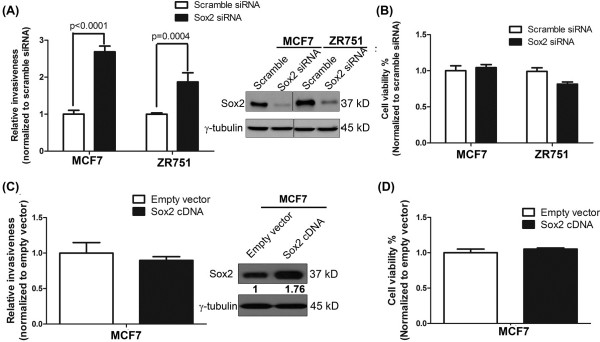
**Sox2 suppresses invasiveness in breast cancer cells. (A)** MCF7 and ZR751 cells were treated with Sox2 siRNA before subjecting to invasion assay. siRNA knockdown of Sox2 significantly increased the invasiveness of MCF7 and ZR751cells. A scrambled siRNA sequence was used as a control and results were normalized to the control. Triplicate experiments were performed. A representative experiment is shown (mean ± standard deviation) (n = 3). Statistical significance was determined by Student's T-test. Western blots analysis showed that siRNA knockdown of Sox2 dramatically decreased the expression of Sox2 in MCF7 and ZR751. **(B)** Cell viability was measured by the MTS assay. siRNA knockdown did not significantly change the viability of MCF7 cells. Similar results were obtained from ZR751 cells. Triplicate experiments were performed (mean ± standard deviation) (n = 6). Statistical significance was determined by Student's T-test. **(C)** Enforced expression of Sox2 did not significantly change the invasiveness of MCF7. MCF7 cells transfected with empty vector were used as a control. Triplicate experiments were performed. A representative experiment is shown (mean ± standard deviation) (n = 3). **(D)** Cell viability was measured by the MTS assay. Enforced expression of Sox2 did not significantly change the viability of MCF7 cells. Triplicate experiments were performed (mean ± standard deviation) (n = 6).

Using an *in-vitro* assay, we assessed if Sox2 regulates the invasiveness of two ER + breast cancer cell lines (i.e., MCF7 and ZR751), both of which have shown the highest expression level of Sox2 described in our previous study [39]. As shown in Figure 1A, siRNA knockdown of Sox2 resulted in a significant increase in the invasiveness of MCF7 and ZR751 cells. These changes were not due to a difference in cell growth between cells treated with Sox2 siRNA or scramble siRNA (Figure 1B). In contrast with the findings of another group [38], we found no significant difference in the invasiveness between MCF7 cells transfected with an empty vector or a Sox2 expression vector (Figure 1C-D).

### The suppression of invasiveness by Sox2 is dependent on the status of the Sox2 transcription activity

As Sox2 is a transcription factor, we asked if Sox2 is transcriptionally active in BC cells, and whether the status of its activity has any impact on its effect on the invasiveness in BC.

To assess the Sox2 transcriptional activity, we have employed a previously characterized *Sox2* reporter. The read-out of the reporter is provided by the inclusion of *green fluorescence protein (GFP)*, driven by a *mCMV* promoter [39]. With the *Sox2* reporter employed, we had identified that MCF7 and ZR751 cells are composed of two phenotypically distinct cell subsets that can be separated based on their differential responsiveness to the *Sox2* reporter [39]. Specifically, cells showing Sox2 transcriptional activity are GFP-positive whereas those showing no evidence of Sox2 transcriptional activity are GFP-negative [39]. For the purpose of this study, the former cell population is labeled ‘reporter responsive’ or RR cells and the latter cell population is labeled ‘reporter un-responsive’ or RU cells. To facilitate our studies, we generated stable cell clones expressing the *Sox2* reporter construct. RR and RU cells were further isolated by flow cytometry and cultured separately. As shown in Additional file 1: Figure S1, the RR and RU cells were readily identified using flow cytometry. Cells stably transfected with the *Sox2* reporter that have not been sorted into RR and RU cells are labeled ‘Sox2R’. We have previously excluded the possibility that the absence of GFP expression in RU cells is due to a lack of Sox2 protein as the vast majority of MCF7 and ZR751 cells expressed Sox2 detected by flow cytometry. Furthermore, by subcellular fractionation, we confirmed that Sox2 is present in the nuclei of these cells [39].

**Figure 2:**
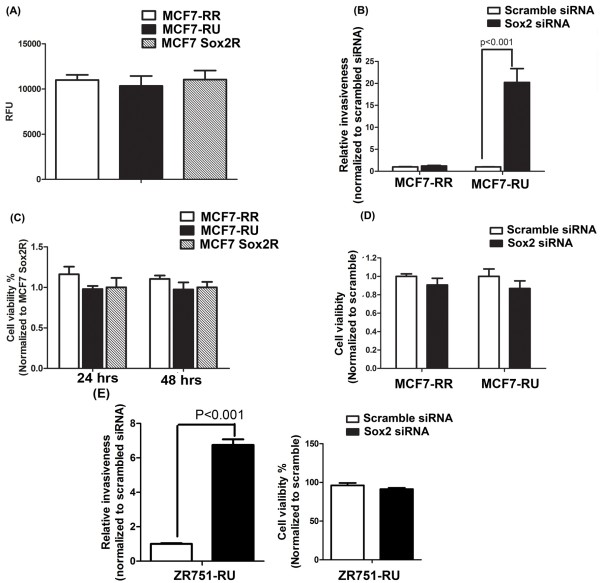
**The suppressive effect of Sox2 on the invasiveness in RU subset but not RR subset. (A)** Cell invasiveness was also assessed using RR cells, RU cells and unsorted cells (labeled as 'Sox2R') derived from MCF7. No significant difference in invasiveness was observed between these three cell populations. Triplicate experiments were performed. A representative experiment is shown (mean ± standard deviation) (n = 3). **(B)** MCF7-RR and -RU cells were subjected to either scramble siRNA or Sox2 siRNA treatment for 24 hour before invasion assay. Sox2 siRNA treatment resulted in significant increase in invasiveness in MCF7-RU cells; no significant change was observed in MCF7-RR cells. Triplicate experiments were performed. A representative experiment is shown (mean ± standard deviation) (n = 3). **(C)** Cell viability of RR, RU, and unsorted cells (labeled as 'Sox2R) from MCF7 were assessed by the MTS assay. **(D)** MCF7-RR and -RU cells were treated with Sox2 siRNA or scramble siRNA before the MTS assay. No significant change in cell viability was found after Sox2 siRNA treatment. **(E)** ZR751-RU cells were subjected to either scramble siRNA or Sox2 siRNA treatment for 24 hour before invasion assay. Sox2 siRNA treatment significantly increases the invasiveness in ZR751-RU cells. Cell viability assay was also performed and no significant change was observed after Sox2 siRNA treatment.

When the invasiveness of RR cells, RU cells and the unsorted Sox2R cells derived from MCF7 was compared, no significant difference was observed among these three cell populations (Figure 2A). However, as shown in Figure 2B, siRNA knockdown of Sox2 resulted in a significant increase in the invasiveness in MCF7-RU cells; in contrast, no significant change was seen in MCF7-RR cells. This difference between the two cell subsets was not due to a significant difference in their cell growth (Figure 2C). In keeping with our previous observation [39], siRNA knockdown of Sox2 also did not result in any significant change in the viability of MCF7-RR and -RU cell populations (Figure 2D). The similar experiments were performed using ZR751-RU cells. In keeping with the results of MCF7 cells, siRNA knockdown of Sox2 in ZR751-RU cells significantly increased in the invasiveness (Figure 2E).

### Sox2 regulates Twist1 expression, but only in RU cells

**Figure 3:**
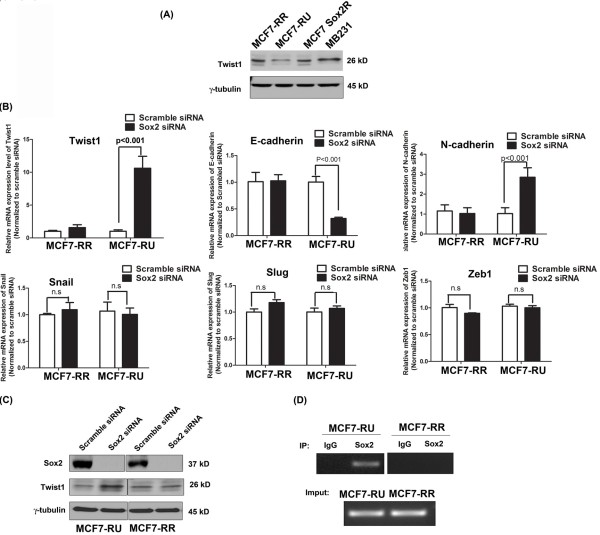
**Modulation of *Twist1 *expression by Sox2 in RU cells but not RR cells. (A)** By western blot analysis, the protein expression of Twist1 was examined in RR, RU and unsorted cells (labeled as 'Sox2R') from MCF7. MB231 was used as positive control. **(B)** MCF7-RR and -RU cells were treated with either scramble siRNA or Sox2 siRNA. By quantitative RT-PCR, the expression level of a panel of EMT/invasiveness inducers were examined, including *Snail1, Slug, ZEB1, MMP2, MMP3, MMP9, Twist1, E-cadherin, N-cadherin, FAK and TGF-β.* siRNA knockdown of Sox2 resulted in significant up-regulation of *Twist1* and *N-cadherin*, down-regulation of *E-cadherin* in MCF7-RU cells but not -RR cells. No significant change was found in the expression level of *Slug*, *Snail*, and *ZEB1, MMP2, MMP3, MMP9, FAK* and *TGF-β* after siRNA knockdown of Sox2. Three representative results (i.e., Slug, Snail, and ZEB1) were shown. Scramble siRNA was used as a control. **(C)** By western blot analysis, the protein expression level of Twist1 was detected after siRNA knockdown of Sox2. **(D)** For the ChIP assay, a normal rabbit IgG antibody or a specific anti-Sox2 antibody was incubated with cross-linked chromatin extracted from MCF7-RR and -RU cells. Isolated DNA was amplified with primer designed against the proximal promoter of *Twist1*. Sox2 was found to bind to the gene promoter region of *Twist1* only in RU but not RR cells. Input control that represents DNA isolated from chromatin before immunoprecipitation shows equal loading. n.s represents no significant difference.

To understand the mechanism by which Sox2 regulates the invasiveness of the RU cells, we examined if Sox2 modulates the expression of factors known to play key roles in regulating the invasiveness and/or EMT in various types of cancers, including Snail1, Slug, ZEB1, MMP2, MMP3, MMP9, Twist1, E-cadherin, N-cadherin, FAK and TGF-β [43-47]. Using quantitative RT-PCR, we found that siRNA knockdown of Sox2 in both MCF7-RR and -RU cells did not result in significant changes in the mRNA levels of *Snail1, Slug, ZEB1* (Figure 3B), as well as *MMP2, MMP3, MMP9, FAK and TGF-β* (not shown). As shown in Figure 3B and C, we found that siRNA knockdown of Sox2 led to a significant up-regulation of the *Twist1* mRNA as well as an upregulation of the Twist1 protein, although these changes were confined to the RU cells. Correlating with these findings, the expres-sion level of *E-cadherin,* one of the key Twist1 downstream targets, was down-regulated in RU cells but not RR cells (Figure 3B). N-cadherin, a cell-cell adhesion mediator, was significantly up-regulated in MCF7-RU cells but not -RR cells. Using ChIP assay, we were able to demonstrate that Sox2 was bound to the promoter region of *Twist1* in RU cells but not RR cells (Figure 3D).

### Modulation of cell invasiveness by Sox2 is mediated via Twist1

**Figure 4:**
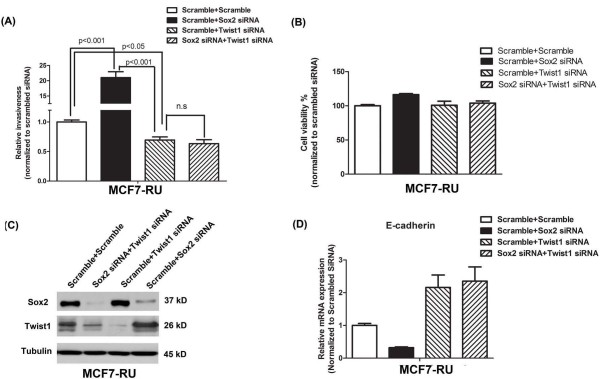
**The role of Sox2 and Twist1 in RU cells. (A)** MCF7-RU cells were subjected to either scramble siRNA, Sox2 siRNA, Twist1 siRNA treatment, or both before cell invasion assay. siRNA knockdown of Sox2 in MCF7-RU cells significantly increased the invasiveness, whereas siRNA knockdown of Twist1 resulted in a significant decrease in invasiveness; Simultaneous knockdown of Sox2 and Twist1 largely abrogated the suppressive effect of Sox2 on invasiveness. Triplicate experiments were performed. A representative experiment is shown (mean ± standard deviation) (n = 3). One-way ANOVA was used to calculated statistics. **(B)** By western blot analysis, Sox2 siRNA and Twist1 siRNA treatment dramatically decreased the expression level of Sox2 and Twist1, respectively. **(C)** Cell viability was measured by the MTS assay. No significant change was observed between the negative control and various treatments. **(D)** By quantitative RT-PCR, the expression level of *E-cadherin* was measured. Cells treated with double scramble siRNA were used as a negative control and data is presented as percentages of control. Statistical significance was determined by one-way ANOVA.

**Figure 5:**
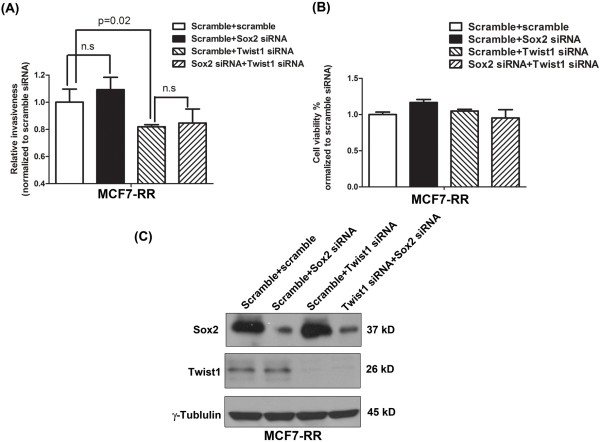
**The role of Sox2 and Twist1 in RR cells.** Similar experiments were performed in MCF7-RU cells as described in Figure 4. **(A)** siRNA knockdown of Sox2 in MCF7-RR cells did not lead to a significant increase in invasiveness. Nevertheless, siRNA knockdown of Twist1 significantly decreased the invasiveness. Cells treated with double scramble siRNA were used as a negative control. Triplicate experiments were performed. A representative experiment is shown (mean ± standard deviation) (n = 3). One-way ANOVA was used to calculated statistics. **(B)** By western blot analysis, Sox2 siRNA and Twist1 siRNA treatment dramatically decrease the expression level of Sox2 and Twist1, respectively. **(C)** Cell viability was measured by the MTS assay. No significant change was observed between the negative control and various treatments.

We then asked if the Sox2-mediated modulation of invasiveness in RU cells is dependent on Twist1. As shown in Figure 4, siRNA knockdown of Sox2 in MCF7-RU cells led to a significant increase in invasiveness, whereas siRNA knockdown of Twist1 led to a significant decrease in invasiveness. Importantly, simultaneous silencing of Sox2 and Twist1 using siRNA largely abrogated the suppressive effect of Sox2 on invasiveness in MCF7-RU cells. These findings strongly suggest that Sox2 suppresses the invasiveness property of RU cells via down-regulating Twist1 in these cells. The same experiment was repeated using MCF7-RR cells and we found no significant change in the invasiveness of these cells (Figure 5). Nevertheless, siRNA knockdown of Twist1 resulted in a significant decrease in the invasiveness of MCF7-RR cells, suggesting that Twist1, but not Sox2, is a key regulator of invasiveness in these cells. Again, the observed differences in invasiveness were not due to a significant difference in the cell growth among the negative controls and various treatment groups (Figure 5B).

## Discussion

The aberrant expression of Sox2 in cancer cells has been found to correlate with the invasiveness of several types of solid tumors [30,34,35,37,48-50]. For instance, a high level of Sox2 expression detectable by immunohistochemistry was found to correlate with higher invasiveness and metastatic potential in gliomas and colorectal cancer [35,36]. Furthermore, siRNA knockdown of Sox2 can result in decreased invasiveness in cell lines derived from gliomas, melanomas and colorectal cancer [35-37]. However, it appears that Sox2 expression in cancer does not always correlate with increased invasiveness and metastasis. We found at least one previous study in which a relatively low level of Sox2 expression in gastric cancer correlates with increased invasiveness/metastatic potential [34]. In the current study, we also found evidence that Sox2 suppresses invasiveness in BC. Thus, the biological effects of Sox2 in cancer cells are likely to be tumor type-specific.

Our finding that Sox2 suppresses the invasiveness of BC is in contrast with that made by another group, who found that enforced expression of Sox2 in MCF7 cells can increases their invasiveness by approximately 60% [38]. In our study, we initially found that siRNA knockdown of Sox2 significantly increased the invasiveness of parental MCF7 cells and MCF7-RU cells. In view of the discrepancy between our conclusion and that described in the literature [38], we attempted to replicate the experiment that examined the effects of enforced Sox2 over-expression in MCF7 cells, as described previously [38], and we did not find any significant change in the invasiveness of these cells (Figure 1C). We would like to point out that the lack of response to enforced Sox2 expression in MCF7 is similar to the finding of one of previous studies, in which enforced expression of Sox2 in MCF7 cells was found to result in no significant change in mammosphere formation and cell growth [39]. While we do not have definitive explanations for the discrepancy between our results and the previously published results [38], we have considered the possibility that the MCF7 cell clones used in the two laboratories may be different. We also have considered the possibility that the *in-vitro* invasiveness assays between the two laboratories have different characteristics. Lastly, since the exact Sox2 protein level has been shown to be functionally important in ESCs [51,52], it is possible that the total Sox2 protein levels after gene transfection are substantially different between the two laboratories, and thus, leading to substantially different biological responses.

The mechanisms by which Sox2 regulate tumor invasiveness have not been extensively studied. In the literature, we were able to identify only 3 studies that are directly relevant to this subject. In all of these three studies (using cell lines derived from colorectal cancer, melanomas and gliomas, respectively), siRNA knockdown of Sox2 was found to decrease invasiveness; in the same three studies, the decrease in invasiveness was found to correlate with a decreased expression level of one of the following molecules: MMP2, MMP3 or FAK [36,37,53]. To our knowledge, the mechanisms by which Sox2 regulates invasiveness in BC are not known. Thus, we screened a panel of factors known to play roles in regulating cell invasiveness/EMT in various types of cancer. In contrast with the previous reports, we did not find any appreciable changes in the expression levels of MMP3, MMP2 and FAK. Instead, we identified Twist1 as the only protein that is regulated by Sox2 in RU cells.

Twist1 has been reported to be one of the master regulators of invasiveness and EMT, and dysregulation of Twist1 expression and function has been implicated to be associated with cancer progression [54-56]. In BC, a high level of Twist1 expression is more common in invasive lobular carcinomas [5]. While siRNA knockdown of Twist1 in BC cells led to a decrease in invasiveness [57], enforced expression of Twist1 in BC cells converts its normal epithelial cell morphology to a spindle-like/fibroblastic morphology [5,58]. In keeping with the concept that Twist1 plays a key role in regulating invasiveness in BC, siRNA knockdown of Twist1 decreased the invasive-ness of both MCF7-RR and -RU cells by approximately 20-30% (Figures 4A and 5A).

As mentioned in the introduction, the expression of Twist1 has been shown to be regulated by a number of proteins such as STAT3, BMP2 and SRC-1. The expression of Sox2 has been shown to correlate with that of Twist1 in human glioblastoma cells [59], although direct proof that Sox2 regulates the expression of Twist1 is lacking. For the first time, we have provided direct evidence that the expression of Twist1 in BC is regulated by Sox2, and this regulation only occurs in the RU cells. Results from our ChIP studies further support the fact that Twist1 is regulated by Sox2 only in RU cells. Although Sox2 does not respond to the reporter in RU cells, possibly due to the fact that Sox2 in RU cells cannot bind to the Sox2 binding motif present in the Sox2 reporter [39], Sox2 in RU cells can bind to the alternative Sox2 binding motif present in the *Twist1* gene promoter and thus suppress its expression as well as invasiveness. These findings are in parallel to the findings that Sox2 is known to negatively regulate a set of genes in ESCs. In contrast, in RR cells, Sox2 does not bind to the promoter region of *Twist1* and the expression of Twist1 is regulated by other factors. The mechanism underlying the decision as to whether Sox2 binds to the *Twist1* gene promoter is under active investigation in our laboratory. Since the transcription activity of Sox2 in normal ESCs has been shown to be modulated by its binding partners, we speculated that a similar scenario may occur in BC cells. Taken together, our findings suggest that the Sox2 transcriptional activity and Twist1 can serve as markers to predict invasiveness in breast cancer cells.

An important concept emerged from the results of this study is related to the significance of the dichotomy of BC cells separated based on the differential responsiveness to the *Sox2* reporter. Specifically, based on our double siRNA knockdown experiments (Figure 4), the Sox2-Twist1 axis plays a key role in regulating the invasiveness in RU cells. In contrast, Twist1, but not Sox2, plays a key role in regulating the invasiveness of RR cells. While the true biological significance of these observations requires further studies, we believe that our results have highlighted a new level of biological complexity of BC. In view of this new knowledge, one may wonder if our current treatments of BC, which are designed based on the assumption that BC cells within a tumor are composed of a biologically uniform population of cancer cells, are fundamentally inadequate. This newly discovered biological complexity of BC cells may prompt us to consider treatment strategies that are based on the recognition of phenotypically distinct cell subsets in BC that are driven by different biochemical pathways.

## Conclusion

In summary, we reported for the first time that Sox2 suppresses invasiveness in BC cells, but only in RU subset. Moreover, Sox2 was found to be a major regulator of Twist1 by controlling the expression level of Twist1. Results from our studies have further supported that the dichotomy of BC based in their differential responsiveness to the *Sox2* reporter carries biological importance, highlighting a new level of biological complexity of BC.

## Abbreviations

Sox2: Sex-determining region Y-box 2; GFP: Green fluorescent protein; ChIP: Chromatin immunoprecipitation; ESC: Embryonic stem cell; BC: Breast cancer; EMT: Epithelial-mesenchymal transition.

## Competing interests

The authors declare that they have no competing interests.

## Authors' contributions

FW and XY performed experiments and analyzed data; PW, KJ, CW, DD, NK, YM and GB assisted with experiments; FW and RL designed the research plan; FW and RL wrote the manuscript. All authors’ read and approved the final manuscript.

## Pre-publication history

The pre-publication history for this paper can be accessed here:

http://www.biomedcentral.com/1471-2407/13/317/prepub

## Supplementary Material

Additional file 1: Figure S1 Identification of the dichotomy of BC cells based on the differential responsivenss to the *Sox2* reporter. (A) MCF7 was stably transfected with either the *Sox2 GFP* reporter or *mCMV* lentiviral vector. Cells stably transfected with the *Sox2 GFP* reporter were labeled as 'MCF7 Sox2R'. Cells stably transfected with *mCMV* control were labeled as 'MCF7 mCMV'. GFP expression was measured by flow cytometry. Cells showing Sox2 transcriptional activity are GFP-positive whereas those showing no evidence of Sox2 transcriptional activity are GFP-negative. For the purpose of this study, the former cell population is labeled ‘reporter responsive’ or RR cells and the latter cell population is labeled ‘reporter un-responsive’ or RU cells. (B) To further examine the biology of these two cell subsets, we isolated and cultured the GFP-positive (labeled as 'RR') and GFP-negative cells (labeled as 'RU') separately from MCF7 cells. Click here for file

## References

[B1] Guarino M Rubino B Ballabio G The role of epithelial-mesenchymal transition in cancer pathology Pathology 2007 39 3 305 318 10.1080/00313020701329914 17558857

[B2] Thiery JP Epithelial-mesenchymal transitions in tumour progression Nat Rev Cancer 2002 2 6 442 454 10.1038/nrc822 12189386

[B3] Bastid J EMT in carcinoma progression and dissemination: facts, unanswered questions, and clinical considerations Cancer Metastasis Rev 2012 31 1–2 277 283 22215472 10.1007/s10555-011-9344-6

[B4] Kalluri R Weinberg RA The basics of epithelial-mesenchymal transition J Clin Invest 2009 119 6 1420 1428 10.1172/JCI39104 19487818 PMC2689101

[B5] Yang J Mani SA Donaher JL Ramaswamy S Itzykson RA Come C Savagner P Gitelman I Richardson A Weinberg RA Twist, a master regulator of morphogenesis, plays an essential role in tumor metastasis Cell 2004 117 7 927 939 10.1016/j.cell.2004.06.006 15210113

[B6] Li QQ Xu JD Wang WJ Cao XX Chen Q Tang F Chen ZQ Liu XP Xu ZD Twist1-mediated adriamycin-induced epithelial-mesenchymal transition relates to multidrug resistance and invasive potential in breast cancer cells Clin Cancer Res 2009 15 8 2657 2665 10.1158/1078-0432.CCR-08-2372 19336515

[B7] Fu J Qin L He T Qin J Hong J Wong J Liao L Xu J The TWIST/Mi2/NuRD protein complex and its essential role in cancer metastasis Cell Res 2011 21 2 275 289 10.1038/cr.2010.118 20714342 PMC3193433

[B8] Cheng GZ Chan J Wang Q Zhang W Sun CD Wang LH Twist transcriptionally up-regulates AKT2 in breast cancer cells leading to increased migration, invasion, and resistance to paclitaxel Cancer Res 2007 67 5 1979 1987 10.1158/0008-5472.CAN-06-1479 17332325

[B9] Vesuna F Van Diest P Chen JH Raman V Twist is a transcriptional repressor of E-cadherin gene expression in breast cancer Biochem Biophys Res Commun 2008 367 2 235 241 10.1016/j.bbrc.2007.11.151 18062917 PMC2696127

[B10] Yang WH Lan HY Huang CH Tai SK Tzeng CH Kao SY Wu KJ Hung MC Yang MH RAC1 activation mediates Twist1-induced cancer cell migration Nat Cell Biol 2012 14 4 366 374 10.1038/ncb2455 22407364

[B11] Tan EJ Thuault S Caja L Carletti T Heldin CH Moustakas A Regulation of transcription factor Twist expression by the DNA architectural protein high mobility group A2 during epithelial-to-mesenchymal transition J Biol Chem 2012 287 10 7134 7145 10.1074/jbc.M111.291385 22241470 PMC3293571

[B12] Hong J Zhou J Fu J He T Qin J Wang L Liao L Xu J Phosphorylation of serine 68 of Twist1 by MAPKs stabilizes Twist1 protein and promotes breast cancer cell invasiveness Cancer Res 2011 71 11 3980 3990 10.1158/0008-5472.CAN-10-2914 21502402 PMC3107354

[B13] Cheng GZ Zhang WZ Sun M Wang Q Coppola D Mansour M Xu LM Costanzo C Cheng JQ Wang LH Twist is transcriptionally induced by activation of STAT3 and mediates STAT3 oncogenic function J Biol Chem 2008 283 21 14665 14673 10.1074/jbc.M707429200 18353781 PMC2386910

[B14] Ma L Lu MF Schwartz RJ Martin JF Bmp2 is essential for cardiac cushion epithelial-mesenchymal transition and myocardial patterning Development 2005 132 24 5601 5611 10.1242/dev.02156 16314491

[B15] Qin L Liu Z Chen H Xu J The steroid receptor coactivator-1 regulates twist expression and promotes breast cancer metastasis Cancer Res 2009 69 9 3819 3827 10.1158/0008-5472.CAN-08-4389 19383905 PMC2911143

[B16] Satoh K Hamada S Kimura K Kanno A Hirota M Umino J Fujibuchi W Masamune A Tanaka N Miura K Up-regulation of MSX2 enhances the malignant phenotype and is associated with twist 1 expression in human pancreatic cancer cells Am J Pathol 2008 172 4 926 939 10.2353/ajpath.2008.070346 18349132 PMC2276419

[B17] Li CW Xia W Huo L Lim SO Wu Y Hsu JL Chao CH Yamaguchi H Yang NK Ding Q Epithelial-mesenchymal transition induced by TNF-alpha requires NF-kappaB-mediated transcriptional upregulation of Twist1 Cancer Res 2012 72 5 1290 1300 10.1158/0008-5472.CAN-11-3123 22253230 PMC3350107

[B18] Kalra J Sutherland BW Stratford AL Dragowska W Gelmon KA Dedhar S Dunn SE Bally MB Suppression of Her2/neu expression through ILK inhibition is regulated by a pathway involving TWIST and YB-1 Oncogene 2010 29 48 6343 6356 10.1038/onc.2010.366 20838384 PMC3007675

[B19] Nairismagi ML Vislovukh A Meng Q Kratassiouk G Beldiman C Petretich M Groisman R Fuchtbauer EM Harel-Bellan A Groisman I Translational control of TWIST1 expression in MCF-10A cell lines recapitulating breast cancer progression Oncogene 2012 31 47 4960 4966 10.1038/onc.2011.650 22266852

[B20] Yang F Sun L Li Q Han X Lei L Zhang H Shang Y SET8 promotes epithelial-mesenchymal transition and confers TWIST dual transcriptional activities EMBO J 2012 31 1 110 123 21983900 10.1038/emboj.2011.364 PMC3252577

[B21] Haque I Banerjee S Mehta S De A Majumder M Mayo MS Kambhampati S Campbell DR Banerjee SK Cysteine-rich 61-connective tissue growth factor-nephroblastoma-overexpressed 5 (CCN5)/Wnt-1-induced signaling protein-2 (WISP-2) regulates microRNA-10b via hypoxia-inducible factor-1alpha-TWIST signaling networks in human breast cancer cells J Biol Chem 2011 286 50 43475 43485 10.1074/jbc.M111.284158 22020939 PMC3234824

[B22] Li S Kendall SE Raices R Finlay J Covarrubias M Liu Z Lowe G Lin YH Teh YH Leigh V TWIST1 associates with NF-kappaB subunit RELA via carboxyl-terminal WR domain to promote cell autonomous invasion through IL8 production BMC Biol 2012 10 73 10.1186/1741-7007-10-73 22891766 PMC3482588

[B23] Eckert MA Lwin TM Chang AT Kim J Danis E Ohno-Machado L Yang J Twist1-induced invadopodia formation promotes tumor metastasis Cancer Cell 2011 19 3 372 386 10.1016/j.ccr.2011.01.036 21397860 PMC3072410

[B24] Keramari M Razavi J Ingman KA Patsch C Edenhofer F Ward CM Kimber SJ Sox2 is essential for formation of trophectoderm in the preimplantation embryo PLoS One 2010 5 11 e13952 10.1371/journal.pone.0013952 21103067 PMC2980489

[B25] Adachi K Suemori H Yasuda SY Nakatsuji N Kawase E Role of SOX2 in maintaining pluripotency of human embryonic stem cells Genes Cells 2010 15 5 455 470 20384793 10.1111/j.1365-2443.2010.01400.x

[B26] Kiefer JC Back to basics: Sox genes Dev Dyn 2007 236 8 2356 2366 10.1002/dvdy.21218 17584862

[B27] Okita K Yamanaka S Induction of pluripotency by defined factors Exp Cell Re 2010 316 16 2565 2570 10.1016/j.yexcr.2010.04.023 20420827

[B28] Park IH Zhao R West JA Yabuuchi A Huo H Ince TA Lerou PH Lensch MW Daley GQ Reprogramming of human somatic cells to pluripotency with defined factors Nature 2008 451 7175 141 146 10.1038/nature06534 18157115

[B29] Annovazzi L Mellai M Caldera V Valente G Schiffer D SOX2 expression and amplification in gliomas and glioma cell lines CANCER GENOMICS PROTEOMICS 2011 8 3 139 147 21518820

[B30] Jia X Li X Xu Y Zhang S Mou W Liu Y Lv D Liu CH Tan X Xiang R SOX2 promotes tumorigenesis and increases the anti-apoptotic property of human prostate cancer cell J Mol Cell Biol 2011 3 4 230 238 10.1093/jmcb/mjr002 21415100

[B31] Chen Y Shi L Zhang L Li R Liang J Yu W Sun L Yang X Wang Y Zhang Y The molecular mechanism governing the oncogenic potential of SOX2 in breast cancer J Biol Chem 2008 283 26 17969 17978 10.1074/jbc.M802917200 18456656

[B32] Chen S Xu Y Chen Y Li X Mou W Wang L Liu Y Reisfeld RA Xiang R Lv D SOX2 gene regulates the transcriptional network of oncogenes and affects tumorigenesis of human lung cancer cells PLoS One 2012 7 5 e36326 10.1371/journal.pone.0036326 22615765 PMC3352903

[B33] Gelebart P Hegazy SA Wang P Bone KM Anand M Sharon D Hitt M Pearson JD Ingham RJ Ma Y Aberrant expression and biological significance of Sox2, an embryonic stem cell transcriptional factor, in ALK-positive anaplastic large cell lymphoma Blood Cancer J 2012 2 e82 10.1038/bcj.2012.27 22885405 PMC3432482

[B34] Zhang X Yu H Yang Y Zhu R Bai J Peng Z He Y Chen L Chen W Fang D SOX2 in gastric carcinoma, but not Hath1, is related to patients' clinicopathological features and prognosis J Gastrointest Surg 2010 14 8 1220 1226 10.1007/s11605-010-1246-3 20532662

[B35] Alonso MM Diez-Valle R Manterola L Rubio A Liu D Cortes-Santiago N Urquiza L Jauregi P Lopez De Munain A Sampron N Genetic and epigenetic modifications of Sox2 contribute to the invasive phenotype of malignant gliomas PLoS One 2011 6 11 e26740 10.1371/journal.pone.0026740 22069467 PMC3206066

[B36] Han X Fang X Lou X Hua D Ding W Foltz G Hood L Yuan Y Lin B Silencing SOX2 induced mesenchymal-epithelial transition and its expression predicts liver and lymph node metastasis of CRC patients PLoS One 2012 7 8 e41335 10.1371/journal.pone.0041335 22912670 PMC3422347

[B37] Girouard SD Laga AC Mihm MC Scolyer RA Thompson JF Zhan Q Widlund HR Lee CW Murphy GF SOX2 contributes to melanoma cell invasion Lab Invest 2012 92 3 362 370 10.1038/labinvest.2011.188 22184093 PMC3887365

[B38] Simoes BM Piva M Iriondo O Comaills V Lopez-Ruiz JA Zabalza I Mieza JA Acinas O Vivanco MD Effects of estrogen on the proportion of stem cells in the breast Breast Cancer Res Treat 2011 129 1 23 35 10.1007/s10549-010-1169-4 20859678

[B39] Wu F Zhang J Wang P Ye X Jung K Bone KM Pearson JD Ingham RJ McMullen TP Ma Y Identification of two novel phenotypically distinct breast cancer cell subsets based on Sox2 transcription activity Cell Signal 2012 24 11 1989 1998 10.1016/j.cellsig.2012.07.008 22800865

[B40] Wu F Wang P Young LC Lai R Li L Proteome-wide identification of novel binding partners to the oncogenic fusion gene protein, NPM-ALK, using tandem affinity purification and mass spectrometry Am J Pathol 2009 174 2 361 370 10.2353/ajpath.2009.080521 19131589 PMC2630546

[B41] Wu F Wang P Zhang J Young LC Lai R Li L Studies of phosphoproteomic changes induced by nucleophosmin-anaplastic lymphoma kinase (ALK) highlight deregulation of tumor necrosis factor (TNF)/Fas/TNF-related apoptosis-induced ligand signaling pathway in ALK-positive anaplastic large cell lymphoma Mol Cell Proteomics 2010 9 7 1616 1632 10.1074/mcp.M000153-MCP201 20393185 PMC2938097

[B42] Zhang J Wang P Wu F Li M Sharon D Ingham RJ Hitt M McMullen TP Lai R Aberrant expression of the transcriptional factor Twist1 promotes invasiveness in ALK-positive anaplastic large cell lymphoma Cell Signal 2012 24 4 852 858 10.1016/j.cellsig.2011.11.020 22155737

[B43] Peinado H Olmeda D Cano A Snail, Zeb and bHLH factors in tumour progression: an alliance against the epithelial phenotype? Nat Rev Cancer 2007 7 6 415 428 17508028 10.1038/nrc2131

[B44] Alkatout I Wiedermann M Bauer M Wenners A Jonat W Klapper W Transcription factors associated with epithelial-mesenchymal transition and cancer stem cells in the tumor centre and margin of invasive breast cancer Exp Mol Pathol 2012 10.1016/j.yexmp.2012.09.003 22985790

[B45] Barrallo-Gimeno A Nieto MA The Snail genes as inducers of cell movement and survival: implications in development and cancer Development 2005 132 14 3151 3161 10.1242/dev.01907 15983400

[B46] Walsh LA Damjanovski S IGF-1 increases invasive potential of MCF 7 breast cancer cells and induces activation of latent TGF-beta1 resulting in epithelial to mesenchymal transition Cell Commun Signal 2011 9 1 10 10.1186/1478-811X-9-10 21535875 PMC3104381

[B47] Wang X Lu H Urvalek AM Li T Yu L Lamar J DiPersio CM Feustel PJ Zhao J KLF8 promotes human breast cancer cell invasion and metastasis by transcriptional activation of MMP9 Oncogene 2011 30 16 1901 1911 10.1038/onc.2010.563 21151179 PMC3952074

[B48] Xiang R Liao D Cheng T Zhou H Shi Q Chuang TS Markowitz D Reisfeld RA Luo Y Downregulation of transcription factor SOX2 in cancer stem cells suppresses growth and metastasis of lung cancer Br J Cancer 2011 104 9 1410 1417 10.1038/bjc.2011.94 21468047 PMC3101944

[B49] Matsuoka J Yashiro M Sakurai K Kubo N Tanaka H Muguruma K Sawada T Ohira M Hirakawa K Role of the stemness factors sox2, oct3/4, and nanog in gastric carcinoma J Surg Res 2012 174 1 130 135 10.1016/j.jss.2010.11.903 21227461

[B50] Sanada Y Yoshida K Ohara M Oeda M Konishi K Tsutani Y Histopathologic evaluation of stepwise progression of pancreatic carcinoma with immunohistochemical analysis of gastric epithelial transcription factor SOX2: comparison of expression patterns between invasive components and cancerous or nonneoplastic intraductal components Pancreas 2006 32 2 164 170 10.1097/01.mpa.0000202947.80117.a0 16552336

[B51] Kopp JL Ormsbee BD Desler M Rizzino A Small increases in the level of Sox2 trigger the differentiation of mouse embryonic stem cells Stem Cells 2008 26 4 903 911 10.1634/stemcells.2007-0951 18238855

[B52] Chew JL Loh YH Zhang W Chen X Tam WL Yeap LS Li P Ang YS Lim B Robson P Reciprocal transcriptional regulation of Pou5f1 and Sox2 via the Oct4/Sox2 complex in embryonic stem cells Mol Cell Biol 2005 25 14 6031 6046 10.1128/MCB.25.14.6031-6046.2005 15988017 PMC1168830

[B53] Oppel F Muller N Schackert G Hendruschk S Martin D Geiger KD Temme A SOX2-RNAi attenuates S-phase entry and induces RhoA-dependent switch to protease-independent amoeboid migration in human glioma cells Mol Cancer 2011 10 137 10.1186/1476-4598-10-137 22070920 PMC3228695

[B54] Hoek K Rimm DL Williams KR Zhao H Ariyan S Lin A Kluger HM Berger AJ Cheng E Trombetta ES Expression profiling reveals novel pathways in the transformation of melanocytes to melanomas Cancer Res 2004 64 15 5270 5282 10.1158/0008-5472.CAN-04-0731 15289333

[B55] Zhang Z Xie D Li X Wong YC Xin D Guan XY Chua CW Leung SC Na Y Wang X Significance of TWIST expression and its association with E-cadherin in bladder cancer Hum Pathol 2007 38 4 598 606 10.1016/j.humpath.2006.10.004 17258791

[B56] Watson MA Ylagan LR Trinkaus KM Gillanders WE Naughton MJ Weilbaecher KN Fleming TP Aft RL Isolation and molecular profiling of bone marrow micrometastases identifies TWIST1 as a marker of early tumor relapse in breast cancer patients Clin Cancer Res 2007 13 17 5001 5009 10.1158/1078-0432.CCR-07-0024 17785550 PMC2680916

[B57] Banerjee A Wu ZS Qian P Kang J Pandey V Liu DX Zhu T Lobie PE ARTEMIN synergizes with TWIST1 to promote metastasis and poor survival outcome in patients with ER negative mammary carcinoma Breast Cancer Res 2011 13 6 R112 10.1186/bcr3054 22060274 PMC3326554

[B58] Mironchik Y Winnard PT Jr Vesuna F Kato Y Wildes F Pathak AP Kominsky S Artemov D Bhujwalla Z Van Diest P Twist overexpression induces in vivo angiogenesis and correlates with chromosomal instability in breast cancer Cancer Res 2005 65 23 10801 10809 10.1158/0008-5472.CAN-05-0712 16322226 PMC5575828

[B59] Velpula KK Dasari VR Tsung AJ Dinh DH Rao JS Cord blood stem cells revert glioma stem cell EMT by down regulating transcriptional activation of Sox2 and Twist1 Oncotarget 2011 2 12 1028 1042 22184289 10.18632/oncotarget.367 PMC3282065

