# Supplementary figures and images for: Sox2 suppresses the invasiveness of breast cancer cells via a mechanism that is dependent on Twist1 and the status of Sox2 transcription activity
Source: BMC Cancer. 2013 Jul 1;13:317. doi: 10.1186/1471-2407-13-317 (PMC3707762; doi:10.1186/1471-2407-13-317)

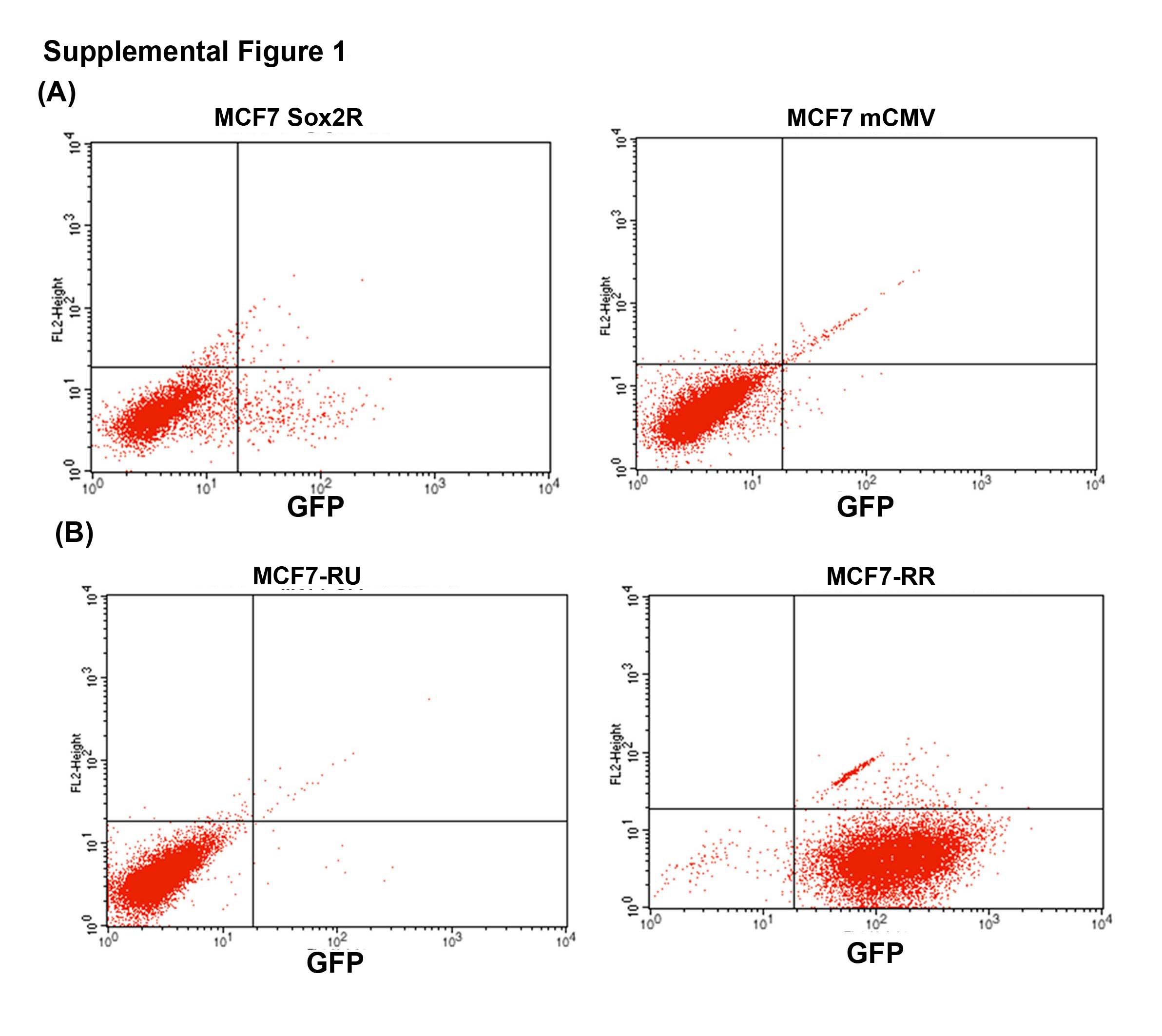

Supplement: Additional file 1: Figure S1 — Identification of the dichotomy of BC cells based on the differential responsivenss to the Sox2 reporter. (A) MCF7 was stably transfected with either the Sox2 GFP reporter or mCMV lentiviral vector. Cells stably transfected with the Sox2 GFP reporter were labeled as 'MCF7 Sox2R'. Cells stably transfected with mCMV control were labeled as 'MCF7 mCMV'. GFP expression was measured by flow cytometry. Cells showing Sox2 transcriptional activity are GFP-positive whereas those showing no evidence of Sox2 transcriptional activity are GFP-negative. For the purpose of this study, the former cell population is labeled ‘reporter responsive’ or RR cells and the latter cell population is labeled ‘reporter un-responsive’ or RU cells. (B) To further examine the biology of these two cell subsets, we isolated and cultured the GFP-positive (labeled as 'RR') and GFP-negative cells (labeled as 'RU') separately from MCF7 cells. [file 1471-2407-13-317-S1.jpeg]
